# Participating in Online Mental Health Interventions: Who Is Most Likely to Sign Up and Why?

**DOI:** 10.1155/2014/790457

**Published:** 2014-04-02

**Authors:** Dimity A. Crisp, Kathleen M. Griffiths

**Affiliations:** ^1^Centre for Mental Health Research, The Australian National University, Canberra, ACT 0200, Australia; ^2^Centre for Applied Psychology, University of Canberra, Canberra, ACT 2601, Australia

## Abstract

Internet-based interventions are increasingly recognized as effective in the treatment and prevention of mental disorders. However, little research has investigated who is most likely to participate in intervention trials. This study examined the characteristics of individuals interested in participating in an online intervention to improve emotional well-being and prevent or reduce the symptoms of depression, factors reported to encourage or discourage participation, and preferences for different intervention types. The study comprised 4761 Australians participating in a survey on emotional health. Comparisons are made between those who expressed an interest in participating in the trial and those who were not. Compared to those who declined to participate, interested participants were more likely older, females, separated/divorced, and highly educated, have reported current or past history of depression, report higher depressive symptoms, and have low personal stigma. Despite the flexibility of online interventions, finding time to participate was the major barrier to engagement. Financial compensation was the most commonly suggested strategy for encouraging participation. An increased understanding of factors associated with nonparticipation may inform the design of future e-mental health intervention trials. Importantly, consideration needs to be given to the competing time pressures of potential participants, in balance with the desired study design.

## 1. Introduction

Depression is one of the major causes of the burden of disease worldwide [1]. Significantly, a large proportion of people with depression do not seek professional help for their condition or find that the services they receive do not adequately meet their needs [2]. This treatment gap has been attributed to a number of factors including the availability and accessibility of services, the stigma associated with depression, and a belief in self-reliance [3, 4].

Internet interventions can offer a nonthreatening avenue to psychological help and a low cost alternative to the traditional mental health system. The Internet also offers the advantage of convenience with greater accessibility and flexibility to extend service delivery to reach a wider population or demographic (e.g., [5, 6]) and provide support to persons who may otherwise be unable to attend face-to-face sessions with a therapist [5, 7–9]. Importantly, there is growing evidence for the efficacy of online interventions for mental health conditions such as depression. Internet-based psychoeducation and cognitive behaviour therapy programs are effective in increasing depression literacy, reducing dysfunctional thinking and depressive symptoms [5, 10–16], and decreasing stigma [13, 17]. Meta-analysis suggests that the effectiveness of online programs is comparable to traditional face-to-face treatment delivery [10]. In addition, Internet-based interventions focusing on facilitating peer support groups may be a promising avenue for providing support to individuals with a mental health condition. A Randomised Controlled Trial (RCT) conducted by Griffiths et al. [18] found that exposure to a 12-week Internet Support Group (ISG) intervention was associated with a reduction in depressive symptoms at 6 and 12 months after intervention relative to an attention control.

With the growth in Internet-based health interventions, research has begun to focus on identifying characteristics of those people who access these service options and who participate in the trials which investigate intervention efficacy. However, the available data is limited and inconsistent. Some research suggests that online interventions or ISGs for mental health problems attract participants who report high levels of psychological distress that are otherwise untreated [19, 20]. However, Houston et al. [21] reported the majority of respondents to a survey of depression ISG users were also receiving other formal treatments (e.g., medication or psychological) but were otherwise socially isolated.

The investigation of other individual and demographic differences has also produced mixed results. Klein and Cook [22] found no significant demographic differences between individuals who preferred using e-mental health services and those with a preference for traditional face-to-face treatment. However, a preference for using e-services was associated with a higher level of stigma, the belief that chance determines an individual's mental health status, and the perception that doctors or other professionals have little influence on a person's mental health. Donkin et al. [23] explored factors associated with the self-selection of participants meeting inclusion criteria for participation in an Internet depression trial as a means of establishing sampling bias in their study. They found that being female, English-speaking, having higher education, and having a prior diagnosis of depression or anxiety were associated with participation.

The strategies employed in promoting an intervention trial are likely to influence the effectiveness of participant recruitment. There is some evidence concerning the factors which encourage or discourage people from participating in e-mental health services. Broadly, ease and convenience of use, and perceived helpfulness of the information or intervention, have been nominated as the principle benefits of online interventions and are reported to underlie user preference for engaging with online interventions [24, 25]. In addition, it has been suggested that opportunities for personal support by means of an incorporated support group or bulletin board are desired by users [21, 24, 26]. However, little research has focused on the factors that predict enrolment or that would encourage future participation in research trials of these interventions.

We recently conducted a large community survey which investigated the emotional health of Australians in city and rural areas and invited respondents to the survey to participate in a randomised controlled trial investigating the usefulness of self-help Internet programs for improving emotional well-being and preventing or reducing the symptoms of depression [18, 27]. The current study has two aims. First to identify the predictors of interest in participating in the online trial, and to examine the survey respondents' reasons for nonparticipation together with their suggestions for encouraging participation in the future. Given the particular importance of understanding factors which are most likely to encourage the participation of those in most need due to high levels of psychological distress, analyses separately examined the pattern of findings for the full sample of respondents and those meeting eligibility criteria for inclusion in the trial. A second aim of the study was to examine the intervention preferences of eligible respondents who volunteered to participate in the study.

## 2. Materials and Methods

### 2.1. Participants and Procedure

**Figure 1:**
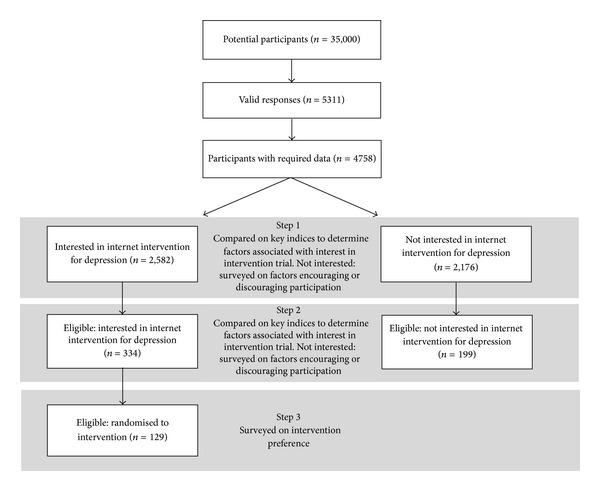
Flow diagram illustrating participant selection.

This study comprised participants from one of the two waves of the Well-Being Project [18, 27]. The second wave of the Well-Being Project was utilised in the present study as, following a lower than anticipated recruitment to the trial in wave 1 of the project, wave 2 had incorporated questions designed to determine survey respondents' reasons for not volunteering for the online trial. A sample of 35,000 individuals aged 18–65, randomly selected for the Australian Electoral Roll, were mailed invitations to participate in a large survey of the emotional health of Australians. A total of 5311 individuals across 2 rural and 2 metropolitan electorates completed the survey (response rate 15.2%). Of the respondents, 550 participants were excluded from analysis because their lack of internet access rendered them ineligible to participate in the trial, or as a result of failing to answer key demographic questions, or indicate whether they would consider participating in the online trial (see flow diagram in Figure 1). Part 1 of this paper reports findings for the remaining 4758 survey respondents.

Following return of the survey, responses were screened for eligibility for the intervention trial. Inclusion criteria for the trial were that participants should be aged between 18 and 65 years, have a K10 score greater than 22, and have home or work access to the internet. Participants were excluded if they were currently receiving Cognitive Behaviour Therapy or other treatment from a mental health professional, were members of a mutual support group at the time of recruitment, or had a self-reported current or past experience or diagnosis of psychosis, schizophrenia, or bipolar disorder. Based on these criteria 533 respondents were identified as eligible for inclusion in the trial. Of the 533 participants who satisfied the inclusion criteria, 334 individuals expressed an interest in participating; however, only 129 formally consented to participating and to a brief phone survey prior to being randomised into the intervention.

Ethics approval was obtained for the study from the Australian National University Committee for Ethics in Human Research (Protocol 2007/2259).

### 2.2. Measures

The survey was conducted via self-report questionnaires mailed to respondents. The survey incorporated a range of psychological and other measures (see [27]). The following demographic characteristics and indicators of psychological distress and stigma are the focus of the present paper.

#### 2.2.1. Demographic Characteristics

Sociodemographic information was obtained relating to age, gender (male = 0, female = 1), marital status (married/de-facto = 0, separated/divorced = 1, widowed = 2, never married = 3), and years of education (0 = less than 15 years, 1 = 15+ years).

#### 2.2.2. Experience of Depression

Past and current experience with depression were assessed using two individual items: “Do you currently suffer from Depression?” and “Have you suffered from depression at any time in your life?” Each item was responded to in a yes/no/maybe format. Categories were then dichotomised into 0 (*no*) and 1 (*yes/maybe*).

#### 2.2.3. Current Psychological Distress

Psychological distress was assessed using the 10-item Kessler Psychological Distress Scale (K10; [28]). Items examined how often in the past 30 days the respondent had experienced a symptom of distress. Items were responded to on a 5-point scale from 1 (*all of the time*) to 5 (*none of the time*). Total scores range from 10 to 50, with a higher score indicating a greater level of distress.

#### 2.2.4. Stigma

Personal stigma toward depression was assessed using the 9-item personal subscale of the Depression Stigma Scale (DSS-Personal; [13, 29]). The scale assesses respondent's personal, negative attitudes toward depression. Items include “Depression is a sign of personal weakness” and “People with depression should snap out of it.” Items were rated on a 5-point scale from 1 (*strongly agree*) to 5 (*strongly disagree*). Total scores range from 0 to 36, a higher score indicative of a greater level of stigma. The scale demonstrated good internal reliability and test-retest reliability with community samples (e.g., [13, 29]).

#### 2.2.5. Consideration Given to Research Participation

Enclosed with the initial Well-Being Project survey, participants received a brochure explaining the nature of the project, who was conducting the study, and why the study was important. This brochure also specifically explained the online intervention trial which was named “The Well-Being Promotion Study.” Upon completing the survey participants responded to a single item, “Would you consider participating in our Well-Being Promotion project?” indicating either yes/no/maybe. Responses were then dichotomised into 0 (*no*) and 1 (*yes/maybe*). Those participants indicating they would not consider participation were then asked to respond to two open-ended items for the purpose of informing future studies. “Could you tell us the main reason why you do not wish to participate in the Well-Being Promotion project?” and “What if anything would make you more interested in participating in the Well-Being Promotion study?”

#### 2.2.6. Intervention Preference

Prior to being randomised to one of the four intervention conditions participants received a telephone call in which the randomised nature of the trial was explained. They were then asked to indicate, if they had a choice, which of the following interventions they would choose: (1) an Internet-based program which asks you to think and read about lifestyle and environmental factors that could improve your well-being and decrease your risk of stress and depression* (HealthWatch);* (2) an Internet-based program which provides information and teaches skills for improving well-being and preventing depression and stress* (E-couch)*; (3) an online support group for improving well-being and coping with stress and depression* (ISG)*; (4) a combination of the skills program and an online support group. Participants were able to indicate more than one preference. Preferences were not ranked.

### 2.3. Statistical Analyses

As the proportion of missing data within the data set was low (<3%), missing data for continuous variables was imputed using the maximum likelihood estimation via the SPSS EM algorithm [30]. A series of chi-square and Student's *t*-tests were conducted to investigate the univariate associations between interest in participating and demographic, depression, and stigma variables. This was followed by a logistic regression analysis to identify predictors of considering participation (reference category = no). Interquartile odds ratios, representing the odds of respondents at the 25th compared to the 75th percentile, were calculated for continuous variables (age, depression score, and stigma) [31]. Analyses were undertaken using STATA Version 10 and PASW19 (IBM SPSS Statistics, 19.0.0.1).

Responses to the open-ended questions were subjected to a qualitative thematic analysis. Two raters independently coded each response using NVivo8 qualitative data analysis software [32]. Agreement between rater coding was 81.7% for reasons for not participating and 92.6% for suggestions regarding encouraging future participation. Discrepancies were subsequently resolved by discussion between the two raters. The responses of each individual could reflect multiple themes.

## 3. Results

### 3.1. Predicting Participation

Demographic characteristics of both the entire sample and those eligible participants who were and were not interested in participating in the study are displayed in Table 1. Respondents ranged in age from 18 to 65 years (M = 44.4, SD = 12.8), were predominantly females (62.6%), and married (71.3%), with almost half reporting having experienced depression at some time in their life.

Univariate analyses of data for the entire sample of 4758 participants indicated that the group interested in participating in the online intervention (54.3% of respondents) were older (*t*(4756) = 3.52, *P* < 0.001; Cohen's *d* standardised mean effect size = 0.10), had significantly higher levels of depression (*t*(4754.42) = 11.22, *P* < 0.001; Cohen's *d* = 0.33), and significantly lower levels of personal stigma (*t*(4756) = −7.61, *P* < 0.001; Cohen's *d* = 0.22). Comparing the demographic details of each group, females were more likely to express an interest in participating in comparison to males (OR = 1.28); persons indicated as separated/divorced (OR = 1.93) or widowed (OR = 1.84) were more likely to express interest in participating than married persons; and persons with greater than 15 years education were more likely to express interest than those with less education (OR = 1.22). Those who had experienced depression at some time in their life (OR = 2.08) and those currently suffering depression (OR = 2.35) were also more likely to indicate interest in participating in the online intervention.

A logistic regression analysis (Table 2) showed that respondent experience with depression and current symptom level predicted interest in participating in the online study such that those indicating they had experienced depression at some time in their life or that they currently suffer from depression were 54% and 32% more likely to indicate interest in participating, respectively. Moreover, the objective measure of current psychological distress obtained through the K10 indicated that higher current distress was predictive of interest in participation (OR = 1.26, *P* < 0.001, CI [1.03, 1.05]). In addition, older age, being female, being separated/divorced as compared to married, and having greater than 15 years education predicted interest in participation. Finally, respondents reporting higher stigma were less likely to express an interest in participating in the online project (OR = 0.76, *P* < 0.001, CI [0.95, 0.97]). However, the model only accounted for 5.1% of the variance in participation interest. No significant interactions were found between current depression status, stigma, and age.

### 3.2. Predicting Participation for Eligible Respondents

A further examination was then conducted on the 533 respondents who met the inclusion criteria. Univariate analyses indicated that the eligible respondents who were interested in participating in the online intervention (62.7% of respondents) were older (*t*(531) = 2.09, *P* = 0.037; Cohen's *d* effect size = 0.18) and had significantly lower levels of personal stigma (*t*(531) = −2.88, *P* = 0.004; Cohen's *d* effect size = 0.25). Those who had experienced depression at some time in their life (OR = 1.89) and those currently suffering depression (OR = 1.56) were also more likely to indicate interest in participation in the online intervention. No other demographic differences were found (see Table 1).

The logistic regression analysis (Table 3) indicated that amongst eligible respondents stigma was the only significant predictor of interest in participating in the online study with respondents reporting higher stigma being less likely to express an interest in participating in the online project (OR = 0.75, *P* = 0.014, CI [0.93, 0.99]). The model only accounted for 3.3% of the variance in participation interest. No significant interactions were found between current depression status, stigma, and age.

### 3.3. Factors Discouraging Participation

**Figure 2:**
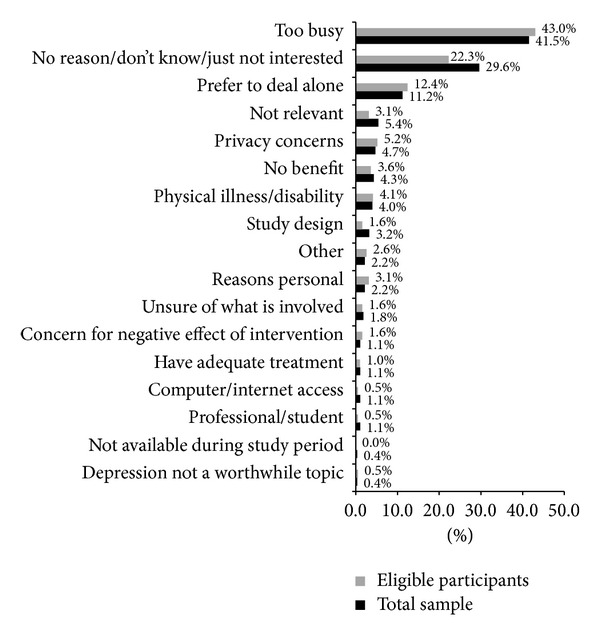
Factors reported as discouraging participation.

Factors discouraging participation were examined both for the total sample of participants who indicated that they would not consider participating in the project and for the subsample of respondents who were eligible but not interested. Of the total sample of 2177 respondents who indicated they would not consider participating in the intervention trial, 277 (13%) reported their reasons for not wanting to participate. In comparison, 193 of the 199 participants (97%) who were eligible, but not willing to participate, reported reasons for their decision. Response themes are summarised for both subsamples in Figure 2. While almost 30% of participants providing data from the full sample and approximately 22% of the eligible respondents indicated “*no reason*” or “*not interested*” in response to why they declined to participate, being “*too busy*” and not having the time to devote to the research study was the most reported theme in both groups (41.5% full sample, 43% eligible respondents). Stigma or embarrassment associated with depression and a preference to deal with problems by oneself and not discuss issues related to depression was also reported by a substantial proportion of respondents (*deal alone:* 11.2% full sample, 12.4% eligible respondents). Other reasons for declining participation included self-exclusion from the study on the basis that they did not believe the program would be relevant for them (*not relevant:* 5.4% full sample, 3.1% eligible respondents), that they had concerns relating to maintaining privacy and confidentiality within the study context (*privacy concerns:* 4.7% full sample, 5.2% eligible respondents), and that the study would not prove any benefit or concern that the intervention would have a negative effect (*lack of confidence:* 4.3% full sample, 3.6% eligible respondents). In addition, a small proportion of people declined as a result of being unsure as to what would be involved in the study, disliking some aspect of the study design, or having adequate treatment or professional knowledge as a student or other healthcare service person (see Figure 2). Furthermore, some participants reported* personal reasons* for not participating including disability or language barriers inhibiting participation.* Other* reasons included practicalities with the timing of the study and unreliable computer or Internet access.

### 3.4. Factors Encouraging Participation in the Future

**Figure 3:**
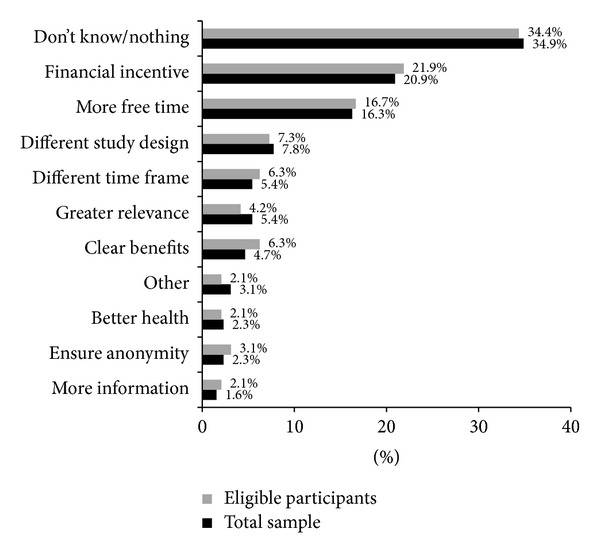
Factors reported as encouraging participation.

Factors that were perceived to encourage participation were examined for both the total sample of participants who indicated that they would not consider participating in the project and for the subsample of those respondents who would otherwise be eligible to participate but were not interested. Of the 2177 people (total sample) who indicated they would not consider participating in the research project, 136 respondents provided information about the factors that would encourage their participation in the future. Ninety-six (of the 199) participants who were eligible, but not interested in participating, provided this information. Response themes are summarised in Figure 3. While approximately 35% of respondents across both samples reported “*nothing*” would encourage their participation, the provision of a “*financial incentive*” was the factor suggested by the greatest proportion of respondents as likely to facilitate their participation (20.9% full sample, 21.9% eligible respondents). In line with the reasons for declining participation, over 16% of respondents noted “*more free time*” would allow their participation or that a* “different study design”* or “*timeframe*” for the study would be more appealing. Other factors highlighted as likely to encourage participation included greater personal* “relevance”* of the study topic. For example, respondents reported that they would be more likely to participate if they had experience with depression or knew someone with depression (5.4% full sample, 4.2% eligible respondents) or if they were able to clearly see either the personal or global benefits of participating in such research (4.7% full sample, 6.3% eligible respondents). The provision of further information concerning the study and the assurance of anonymity for those participating were also suggested. Personal circumstances (e.g.,* better health*) were also noted as contributing to the possibility of future participation (see Figure 3).

### 3.5. Intervention Preference

To investigate participants' preferences for type of intervention we examined the characteristics of the 129 eligible and consenting wave 2 participants prior to their randomisation into the trial. Given the choice (noting that participants could indicate a preference for more than one intervention), the e-couch intervention by itself was indicated as preferred by the greatest proportion of respondents (54.3%), followed by HealthWatch (47.3%), the combined e-couch and ISG (35.7%), and finally the ISG delivered by itself, preferred by only 14.7% of prospective participants. Collapsing across categories, 11% of participants indicated that they were interested in participating in any of the conditions, 77.5% in e-couch either by itself or in combination with the ISG, and 38.8% in the ISG alone or in combination with e-couch.

Univariate analyses indicated no significant effects of age, gender, marital status, past experience of depression, personal stigma, or psychological distress on interest in the different programs offered. However, compared to those not currently depressed, there was a trend for people currently reporting depression to be more likely to express interest in the ISG (76.0% versus 24.0%. *P* = 0.054).

## 4. Discussion

### 4.1. Who Participates in E-Mental Health Programs?

As a means of advancing our understanding and optimising recruitment to online mental health interventions, this study investigated the characteristics of individuals interested in participating in an online well-being and depression intervention and research trial and the factors self-reported to encourage or discourage participation.

We found that a current report of depression or higher psychological distress was associated with a greater interest in participating in the trial. It is encouraging that those most in need of an intervention were those most likely to express an interest in participation. Although only representing small effects, other factors that were independently associated with an interest in participating in the online trial were older age, being female, being separate or divorced, and higher education. These findings are largely consistent with those reported by Donkin et al. [23] who undertook a similar exploration of factors associated with the likelihood of consenting to an online cognitive behavioural therapy program for depression. However, we also demonstrated that a high level of personal stigma was associated with a greater likelihood of refusal to participate. To our knowledge no other research has investigated this question.

It is of particular importance to consider factors which are most likely to encourage or discourage participation among those in most need due to high levels of psychological distress. Accordingly, we examined the characteristics of these “eligible” individuals and found that stigma was the only significant predictor of interest in participating in an online trial. Again, a high level of personal stigma was associated with a greater likelihood of refusal to participate.

One of the principle advantages of Internet-based interventions is their potential for delivering help anonymously and privately. Indeed, Klein and Cook [22] found that a high level of stigma was related to a preference for e-services. Nevertheless, the current findings suggest that personal stigma may be a barrier to help-seeking even in an online environment. Alternatively, or in addition, the findings may reflect a lack of willingness among those with higher levels of stigma to participate in the* research* component of the study. The data does not separate out the contribution of stigma to participant interest in research as opposed to intervention participation. Further quantitative studies are required to distinguish between these two possibilities. An examination of reasons for nonparticipation highlights privacy concerns held by some prospective participants which may align with the effect for stigma. Although personalisation of intervention delivery and the provision of automatic or password protected access can help alleviate concerns and barriers to participation relating to privacy [33], perceived vulnerabilities in Internet security systems or uncertainties around the use or storage of personal data may discourage participation both in online programs and in online research trials. While every effort was made in the present study to assure potential participants that the privacy of their data would be protected and of the strict guidelines that would be followed to ensure this, privacy was nevertheless cited as a concern by several people. This suggests that methods for increasing participant confidence in this aspect of trial implementation should be considered further in developing future recruitment strategies for online studies.

### 4.2. Reported Barriers and Future Incentives

The greatest barrier to participation in the present study appeared to be time. Information provided to prospective participants of the current study stated that the intervention program (The Well-Being Promotion Study) would last 12 weeks and requires approximately half an hour of participants' time each week. Across both the total sample and subsample of eligible participants, over 40% of respondents indicated that they were too busy to participate in such a program and that they would require greater flexibility in their daily schedule to afford them the time to engage in such a task. This raises an important question. Those who promote Internet-based interventions typically emphasise their convenience, accessibility, and flexibility as a means of delivering mental health care and support. Nevertheless, the present study suggests that many individuals, even in psychological distress, express the view that they cannot afford the time in their busy schedules to avail themselves of an online intervention. Indeed, while our initial analyses demonstrated that those respondents who self-reported current depression were more likely to report an interest in participating in general, our investigation based on levels of psychological distress indicated this was not a predictor of interest in participation, and many otherwise eligible participants reported time constraints as a reason for declining to be involved. This suggests that we should investigate methods for reducing the length of online interventions while not compromising their effectiveness. Specifically, we acknowledge that the 12-week intervention period promoted in the present study was longer than most [11].

Further factors such as being “unsure of what is involved” and “concern for negative effects,” although only reported by a small number of participants, are consistent with findings reported by Hoek and colleagues [34] who also reported “not knowing what to expect” as a determinant of nonparticipation. This emphasises the importance of clearly communicating information about the nature and efficacy on online programs. Perhaps recruitment rates in the present study would have been higher if we had supplied additional information about the type of self-help Internet programs that would comprise the intervention. However, this must be balanced against the possibility that too much information might overload the individual and deter participation.

The factors which were identified as encouraging participation were consistent with the factors that individuals cited as discouraging. In particular, greater personal relevance, clear benefits, and a greater time to dedicate to such an endeavour were reported as factors that would increase participation. In addition, financial incentives were proposed by over 20% of respondents. Some past research (e.g., [35]) has found evidence to support the use of small cash incentives to improve enrolment and retention in online programs. However, other researches have failed to find any substantial positive impact of financial incentives on participation (e.g., [36, 37]). Further research is required to explore the potential benefits of delivering financial incentives, and what level of financial incentive might be useful, if any. There is also a need to consider the potential barriers, including ethical issues associated with the use of financial incentives.

### 4.3. Intervention Preference

In examining the question of intervention preference and the type of program desired by participants our study found a strong interest, by over three quarters of our sample, in an opportunity to participate in the e-couch program, an interactive psychological Internet-based intervention which provides information and teaches skills for improving well-being and preventing depression and stress. Cognitive behaviour therapy (CBT) is a commonly used therapeutic technique in online depression interventions (e.g. [12, 14]). The interest in this type of intervention by the majority of participants is therefore encouraging. While current research into the perceptions of users regarding the acceptability of computerised CBT is limited (e.g., [38]), this study does lend further support for the use of this type of program as a basis for mental health care in an online environment.

“HealthWatch,” the attention control for trial, was nominated as a desired treatment by over 47% of participants. This provides evidence of the face validity of this control. However, promoted as “an Internet-based program focused on lifestyle and environmental factors that could improve your wellbeing and decrease your risk of stress and depression,” it may also reflect a desire and preference among the population for access to broad health and well-being initiatives that extend beyond closely targeted illness specific programs. The current findings raise the possibility that a holistic and positive approach to mental and physical health is desired by consumers with elevated levels of psychological distress. The current finding is also consistent with one study in which a strong preference was reported for a control intervention involving weekly telephone calls to discuss lifestyle and environmental factors that may have an influence on depression [39].

Finally, 38.8% of participants were interested in the internet support group (ISG). While studies suggest that ISGs are similar to face-to-face support groups with respect to the supportive environment they provide [7] and depression ISGs are widely used [40], this lower level of interest in the ISG may reflect that acceptability of e-mental health services varies across intervention types. While a preference for online social interaction has been found to be associated with higher levels of depression and loneliness [41] and in the present study participants currently reporting depression were more likely to express an interest in the ISG, further research is required to investigate if support groups, regardless of modality (Internet or face-to-face), are somewhat less preferred than other types of interventions.

## 5. Limitations 

These findings are presented in the context of several limitations. A significant proportion of people did not return “The Well-Being Survey.” Thus, our analysis of the predictors of interest in participation was limited to that group of participants who were willing to complete a survey of emotional well-being but were unwilling to participate in an online trial. This group may not be representative of the broader population of participants who failed to participate in the survey. Further, of those who completed the survey and indicated an unwillingness to participate in the trial, only a minority completed the open-ended question reporting the reasons for their unwillingness to participate and strategies which might increase participation. It is therefore not possible to assess how programs may reach distressed individuals from this group. Finally, as noted previously, it is not possible in the context of the present study to separate an unwillingness to seek any treatment, or specifically treatment online, from a refusal to participate in a research trial. However, to the best of our knowledge the study represents one of the first investigations into the reasons for nonparticipation in an RCT, particularly for an online intervention, from a sample of members of the community.

## 6. Conclusions

The Internet has become an increasingly popular avenue for people to access mental health information, services, and treatment. As such, it is critical that we continue to grow the body of empirical evidence investigating the efficacy of online interventions for mental health conditions such as depression. However, in doing so it is important that consideration is given to the representativeness and the characteristics of trial participants as well as the potential barriers that may exist for participation in online interventions more generally. Specifically, this study highlights a significant association between personal stigma and refusal to participate in an online trial which is consistent with evidence that stigma is one of the major barriers to the utilisation of services in general [3, 4]. Anonymity and privacy are often promoted as a principle advantage of Internet-based interventions. The present findings do not preclude this or the possibility that stigma is a more powerful barrier to face-to-face than online help seeking. However, the data do suggest the need for further investigation of the nature and role of stigmatising attitudes as a barrier to participating in online programs.

By understanding and more closely examining those factors associated with nonparticipation and factors reported to encourage future participation we may also improve our recruitment rates for online intervention trials. Specifically, it appears that careful consideration should be given to the time commitment required from participants in longitudinal trials and in particular to investigate how to optimise mental health outcomes using brief interventions. Aligned with this, further investigation is required into the use of financial incentives or compensation for participation, examining both the potential benefits and the ethical concerns. Finally, this study has highlighted the importance of ensuring that the information provided in participant recruitment materials provides a clear emphasis on the nature, focus, and importance of the trial and on the protection of anonymity and confidentiality of participants.

## Figures and Tables

**Table 1:** Descriptive statistics by interest in participation with univariate group comparisons.

	Total sample	Interested in participating in online intervention? (Total sample)		*P*	Total eligible participants*	Interested in participating in online intervention? (Eligible participants only)		*P*
		No	Yes/maybe			No	Yes/maybe	
*N*	4758	2176	2582		533	199	334	
Demographics								
Age, M (SD)	44.4 (12.8)	43.70 (12.94)	45.01 (12.56)	<0.001	41.27 (13.41)	39.70 (13.37)	42.20 (13.36)	0.037
Gender				<0.001				0.136
Male (%)	37.3	40.5	34.7		33.3	36.2	29.9	
Female (%)	62.7	59.5	65.3		67.7	63.8	70.1	
Education				0.001				0.340
<15 years (%)	50.9	53.5	48.6		58.2	60.8	56.6	
15+ years (%)	49.1	46.5	51.4		41.8	39.2	43.4	
Marital status				<0.001				0.716
Married/de-facto (%)	71.3	73.7	69.3		58.5	57.8	59.0	
Separated/divorced (%)	8.4	5.8	10.5		11.1	9.5	12.0	
Widowed (%)	1.2	0.8	1.4		1.1	1.0	1.2	
Never married (%)	19.2	19.7	18.7		29.3	31.7	27.8	
Ever experienced depression	49.0	39.2	57.3	<0.001	82.0	75.9	85.6	0.005
Currently suffering depression	19.0	12.2	24.6	<0.001	59.5	52.8	63.5	0.015
Depression score (K10)	16.11 (6.17)	15.05 (5.45)	17.01 (6.59)	<0.001	26.98 (4.92)	26.80 (4.79)	27.08 (5.00)	0.519
Stigma	19.93 (5.57)	20.59 (5.52)	19.36 (5.55)	<0.001	21.85 (5.78)	22.78 (6.17)	21.30(5.47)	0.004

*Note: respondents are deemed eligible based on inclusion and exclusion criteria.

**Table 2:** Predictors of participation interest (*n* = 4758).

	*B*	SE	OR	95% CI	
				Lower	Upper
Intercept	−0.73	0.22	0.48***	0.31	0.74
Age	0.01	0.003	1.27***	1.01	1.02
Female	0.15	0.06	1.17*	1.03	1.32
Education	0.27	0.06	1.31***	1.16	1.48
Marital status					
Separated/divorced	0.41	0.12	1.51***	1.19	1.90
Widowed	0.41	0.30	1.50	0.83	2.70
Never married	0.06	0.09	1.06	0.89	1.27
Ever experienced depression	0.43	0.07	1.54***	1.35	1.76
Currently suffering depression	0.27	0.10	1.32**	1.07	1.61
Depression score (K10)	0.04	0.01	1.26***	1.03	1.05
Stigma	−0.04	0.01	0.76***	0.95	0.97

Note: reference categories for categorical predictors are as follows: female (ref = male), marital status (ref = married/de-facto), education (ref = <15 years), ever experienced depression (ref = no), and currently suffering depression (ref = no).

***P < 0.001, **P < 0.01, *P < 0.05.

**Table 3:** Predictors of participation interest for eligible participants (*n* = 533).

	*B*	SE	OR	95% CI	
				Lower	Upper
Intercept	−0.08	0.79	0.92	0.20	4.29
Age	0.02	0.01	1.41	1.00	1.03
Female	0.17	0.20	1.19	0.80	1.76
Education	0.29	0.19	1.33	0.92	1.93
Marital status					
Separated/divorced	0.17	0.31	1.18	0.64	2.18
Widowed	0.02	0.90	1.02	0.17	5.98
Never married	0.08	0.25	1.08	0.67	1.77
Ever experienced depression	0.37	0.26	1.45	0.87	2.43
Currently suffering depression	0.27	0.22	1.32	0.86	2.01
Depression score (K10)	0.01	0.02	1.04	0.97	1.05
Stigma	−0.04	0.02	0.75*	0.93	0.99

Note: reference categories for categorical predictors are as follows: female (ref = male), marital status (ref = married/de-facto), education (ref = <15 years), ever experienced depression (ref = no), and currently suffering depression (ref = no).

*P < 0.05.
